# Structural Regulation, Photothermal Conversion, and Interfacial Heat Transfer Mechanisms of Silver Nanoparticle/Wood-Derived Porous Carbon Composite Phase Change Materials

**DOI:** 10.3390/nano16120779

**Published:** 2026-06-20

**Authors:** Peilin Cheng, Yafeng Li, Zhiwen Yin

**Affiliations:** 1 School of Artificial Intelligence, Zhejiang Business Technology Institute, Ningbo 315012, China; 2 School of Advanced Manufacturing, Nanchang University, Nanchang 330031, China

**Keywords:** composite phase change materials, wood-derived porous carbon, silver nanoparticle modification, molecular dynamics simulation

## Abstract

To address the application bottlenecks of organic phase change materials characterized by low thermal conductivity and susceptibility to liquid leakage, this study utilized natural poplar wood as a raw material to construct a three-dimensional carbon/silver heterogeneous porous skeleton via delignification, gradient carbonization, and in situ electroless silver plating. Polyethylene glycol (PEG) was then vacuum-encapsulated within this structure to prepare form-stable composite phase change materials (CPCMs). The regulatory effects of carbonization temperature and metal interface modification on the microscopic morphology and thermophysical properties of the materials were systematically investigated. The results indicate that the skeleton carbonized at 800 °C achieves an optimal balance between pore distribution and skeleton rigidity, ensuring the uniform conformal growth of silver nanoparticles and endowing the material with excellent anti-leakage performance. The thermal conductivity of the optimal sample reaches as high as 0.683 W/(m·K), with the melting latent heat maintained at 133.9 J/g, while also demonstrating an agile and stable photothermal conversion response. Non-equilibrium molecular dynamics (NEMD) simulations further confirm that the silver nanoparticle modification layer smooths the phonon vibration frequency mismatch between the carbon substrate and organic segments, significantly reducing the interfacial thermal resistance. This research provides an important reference for the structural design and microscopic heat transfer mechanism analysis of high-performance phase change energy storage materials.

## 1. Introduction

Organic phase change materials (Phase Change Materials, PCMs), such as polyethylene glycol (PEG), exhibit significant application prospects in fields including solar energy utilization and electronic device thermal management due to their high latent heat energy storage density, excellent chemical stability, and adjustable temperature response range [1]. Particularly in the realm of sustainable thermal management, recent comprehensive studies have profoundly highlighted the pivotal role of advanced PCMs in regulating environmental temperatures and reducing energy consumption [2,3]. However, the vast majority of organic PCMs face two inherent defects restricting their large-scale engineering application: an extremely low thermal conductivity (typically below 0.3 W/(m·K)) and a high susceptibility to macroscopic liquid leakage during the solid–liquid phase transition process.

To overcome the aforementioned bottlenecks, encapsulating PCMs within three-dimensional porous support skeletons to construct form-stable composite phase change materials (CPCMs) has become a mainstream strategy [4,5]. Internationally, prominent European research groups, such as Cabeza et al., have made foundational contributions by systematically evaluating the material selection and encapsulation criteria of PCMs for diverse thermal energy storage applications [6]. Building on this framework, researchers have explored various skeleton materials. Luo et al. prepared graphene-doped polyvinyl alcohol (PVA) aerogels through vacuum freeze-drying technology, significantly enhancing the thermal conduction and photothermal conversion efficiency of the composites [7]; Huang et al. developed flexible CPCMs reinforced with expanded graphite, successfully applying them to battery thermal management [8]. Among numerous substrates, wood-derived porous carbon stands out because it inherits the anisotropic hierarchical honeycomb-like pores of natural trees and possesses characteristics such as low density and excellent mechanical support. Highly interconnected wood microchannels can lock liquid PCMs within confined spaces through strong capillary forces. For instance, Xie et al. utilized biological grapefruit peel-derived porous carbon to support silver nanoparticles, substantially enhancing the heat transfer capability of the system while maintaining a high loading capacity [9]. Nevertheless, the intrinsic thermal conductivity of amorphous carbon skeletons obtained through conventional pyrolysis remains limited, and the substantial physical property differences between inorganic carbon substrates and organic PCMs lead to extremely high interfacial thermal resistance (ITR), inducing strong phonon scattering. Fundamental theories on nanoscale thermal transport and interfacial phonon scattering, pioneered by US researchers such as Cahill and Chen [10,11], emphasize that atomic-level interface engineering is essential to bridge the vibrational mismatch and minimize ITR.

Interfacial structural engineering is therefore crucial for breaking through the heat transfer bottleneck. Modifying the surface of carbon skeletons with highly thermally conductive metal nanoparticles (such as elemental silver) is an effective approach to constructing continuous phonon transmission channels [12,13,14]. Chen et al. prepared a melamine resin-based skeleton modified with silver nanoparticles and reduced graphene oxide using a high-temperature one-step reduction method, constructing a high-density three-dimensional thermally conductive network [15]; Luo et al. achieved a synergistic improvement in photothermal capture and thermal conductivity by combining silver nanoparticles with expanded graphite [16]. However, achieving uniform and firm loading of nano-metal particles within complex confined spaces while maintaining the unhindered flow of the original deep and long microchannels of wood remains challenging. In addition, current research predominantly relies on macroscopic thermophysical property characterization. A critical scientific question that remains fundamentally unanswered is how the interfacial metal layer regulates phonon transport modes to smooth the vibrational frequency mismatch and minimize interfacial thermal resistance (ITR) at the atomic scale, as well as how it affects the molecular chain diffusion behavior under nanospace confinement. Revealing these microscopic mechanisms is of vital scientific significance for unlocking the core transport physics of advanced phase change materials. Liu et al. previously revealed the enhancing effect of amino functional groups on the phonon coupling between the UiO-66 and stearic acid interfaces through simulation methods, demonstrating the significant value of microscopic simulations in uncovering interfacial mechanisms [17].

While numerous review studies and experimental papers have comprehensively documented the macroscopic performance enhancements of CPCMs, they predominantly focus on material screening and bulk property characterization. There remains a profound knowledge gap regarding the atomic-level interactions at multi-component heterogeneous interfaces. Therefore, the unique niche of this study lies in bridging macroscopic thermodynamic behaviors with microscale transport physics. Based on this, the present study proposes an innovative strategy of “microstructural optimization and interfacial metal modification” for synergistic enhancement. Using natural poplar wood as the raw material, a three-dimensional network with optimal pore openness and skeleton rigidity is screened out through chemical delignification and gradient temperature-controlled carbonization processes. Subsequently, an ultrasound-assisted wet chemical method is employed to uniformly construct a silver nanoparticle modification layer on the pore wall surfaces, achieving highly efficient encapsulation of PEG. Building upon the preliminary work of Yin et al. [18], the present work systematically evaluates the macroscopic impacts of carbonization temperature and metal modification on the leakage prevention, heat storage, and photothermal conversion efficiency of the composites. Crucially, to answer the aforementioned scientific question, non-equilibrium molecular dynamics (NEMD) simulations are introduced. By calculating the phonon density of states (PDOS) and mean square displacement (MSD), the heat transfer enhancement origin of the silver nanoparticle modification layer in smoothing interfacial phonon mismatch and suppressing phonon scattering is quantitatively revealed at the atomic scale, providing an in-depth explanation for the evolutionary laws of polymer phase change dynamics within confined spaces.

## 2. Experimental Section

### 2.1. Materials

Poplar wood blocks (20 mm × 20 mm × 5 mm) were collected from Hebei. Polyethylene glycol (PEG6000, Mn = 6000), sodium chlorite (NaClO_2_), and glacial acetic acid were purchased from Aladdin Chemical Reagent Co., Ltd. (Shanghai, China). Silver nitrate (AgNO_3_, analytical grade, purity ≥ 99.8%) was acquired from Sinopharm Chemical Reagent Co., Ltd. (Shanghai, China). Polyvinylpyrrolidone (PVP, Mn = 58,000) was purchased from Chengdu Kelong Chemical Reagent Factory (Chengdu, China). All chemical reagents used in this study were of commercially available analytical grade or higher and were used directly without further purification. Deionized water was used for all experiments.

### 2.2. Preparation of Porous Composite Carrier DA

**Figure 1:**
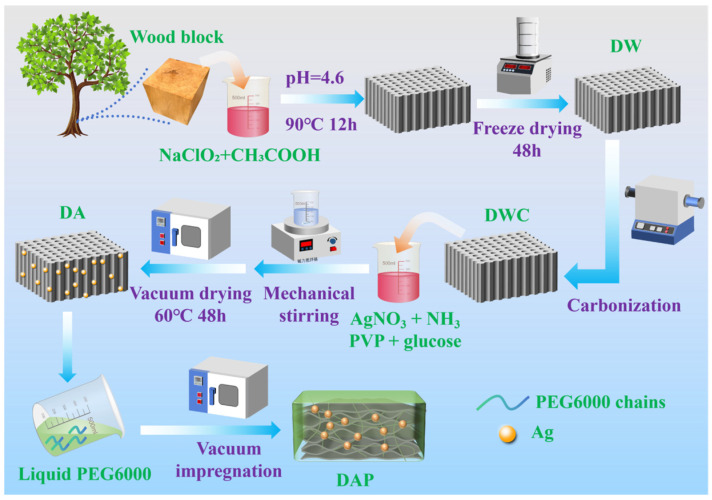
Flowchart of the preparation process of wood-derived functional composite phase change materials.

Figure 1 illustrates the preparation flowchart for the wood-derived functional composite phase change materials. First, pretreated poplar wood blocks were completely immersed in a 2 wt% NaClO_2_ solution. An appropriate amount of glacial acetic acid was added dropwise to stably adjust the pH value of the solution to 4.6, and the reaction system was placed in a 90 °C water bath to react at a constant temperature for 12 h to selectively remove the lignin inside the wood. After the reaction, the samples were subjected to multiple cycles of washing with deionized water, followed by vacuum freeze-drying at −50 °C for 48 h. This yielded delignified wood (DW) that retained its original network structure and well-developed pores.

Subsequently, the DW samples were subjected to structural reforming through a high-temperature carbonization process. The DW samples were placed in a tube furnace and underwent programmed temperature-controlled pyrolysis under a continuous flow of high-purity argon gas for protection. The entire carbonization process was divided into two stages: first, it was slowly heated at a rate of 2 °C/min to 200 °C and kept at a constant temperature for 2 h for pre-carbonization to promote the initial stabilization of the wood skeleton; then, the heating rate was adjusted to 5 °C/min, heating to target final temperatures of 600 °C, 800 °C, and 1000 °C, respectively, and maintaining the temperature for another 2 h for deep carbonization. Corresponding to the different final carbonization temperatures, the obtained samples were named DWC600, DWC800, and DWC1000, respectively.

Finally, a wet chemical method was utilized to construct a silver nanoparticle coating in situ on the inner pore wall surfaces of the DWC. Precisely 3.2 g of AgNO_3_ was weighed and dissolved in water. Then 0.1 mL of ammonia water (28%) was slowly added dropwise along with 0.02 g of PVP as a dispersion stabilizer to prepare a uniform silver-ammonia complex precursor solution. The DWC samples were immersed in this solution and subjected to synergistic ultrasonic and magnetic stirring treatment at a rotation speed of 300 rpm at room temperature for 60 min to ensure that the complex ions were fully adsorbed within the pores. After treatment, the samples were transferred to 50 mL of D-glucose solution (32 g/L), and a reduction reaction was carried out in a 60 °C constant temperature water bath for 4 h. The resulting products were thoroughly washed with deionized water and vacuum-dried under a negative pressure of −0.1 MPa at 60 °C for 48 h, ultimately yielding the structurally complete three-dimensional porous composite carrier DA.

### 2.3. Preparation of Composite Phase Change Material DAP

A vacuum impregnation process was employed to encapsulate the phase change material PEG6000 within the porous DA carrier. First, solid PEG6000 was placed in an 85 °C vacuum drying oven and heated until completely melted. The DA carriers prepared at different carbonization temperatures mentioned above were completely immersed in the molten PEG, and a vacuum pump was activated to reduce the system pressure to below −0.095 MPa, maintaining the vacuum state for 6 h. The synergistic effect of the negative pressure environment and the capillary forces generated by the natural directional pores of the wood strongly drove the low-viscosity liquid PEG to fully infiltrate and fill the micro/nano-pores inside the carrier.

After impregnation, to remove residual PEG on the material surface and further optimize its shape stability, the saturated impregnated samples were taken out. Excess liquid PEG on the surface was initially absorbed with filter paper, and then the samples were placed in an 85 °C atmospheric pressure environment. During this period, the bottom filter paper was replaced every 30 min, and the mass of the sample was accurately recorded. When the continuous weight changes recorded three times were all less than 0.5%, it was considered that the internal adsorption and leakage of PEG had reached a dynamic equilibrium state. Through the above process, composite phase change materials with uniformly distributed PEG and no macroscopic leakage risks were successfully prepared. Corresponding to the aforementioned carbonization temperatures, these composite products were named DAP600, DAP800, and DAP1000, respectively.

### 2.4. Characterization Methods

A field emission scanning electron microscope (FE-SEM, FEI Quanta 118200 FEG, Hill sboro, OR, USA) was used to observe the microscopic structure and morphology of the samples. Fourier transform infrared spectroscopy (FTIR, Nicolet 5700, Madison, WI, USA), X-ray diffraction (XRD, Bruker D8 ADVANCE, Karlsruhe, Germany), and Raman spectroscopy (LabRAM Aramis, Palaiseau, France) were utilized to characterize the structural information of the samples. A differential scanning calorimeter (DSC, NETZSCH STA 449 F5, Selb, Germany) measured the phase change thermal properties in a nitrogen atmosphere at a heating/cooling rate of 2 °C·min^−1^ within a range of 10–90 °C. A thermogravimetric analyzer (TGA, NETZSCH STA 449 F3, Selb, Germany) evaluated the thermal stability in a nitrogen atmosphere at a heating rate of 5 °C·min^−1^ up to a maximum temperature of 600 °C. A transient plane source thermal constant analyzer (Hot Disk 2500-OT, Göteborg, Sweden) was employed to determine the thermal conductivity. The leakage prevention performance was evaluated by heating the samples on a 90 °C heating plate and recording their appearance (digital camera) and surface temperature changes (infrared thermal imager).

To ensure the accuracy and statistical reliability of the experimental data, all key quantitative thermophysical measurements—specifically the thermal conductivity and the latent heat of phase transition—were independently repeated three times for each sample group. The results reported in this study represent the arithmetic average of these measurements, with the corresponding standard deviations expressed as numerical ranges (±SD) in the text or as error bars in the figures.

## 3. Molecular Dynamics Simulation

This study bridges the gap between macroscopic thermophysical properties and atomic-level structural dynamics using molecular dynamics (MD) simulations. While experiments can quantitatively measure improvements in thermal conductivity and shape stability, MD simulations offer unique advantages, providing microscopic insights into underlying physical mechanisms. Specifically, by calculating interfacial thermal resistance (ITR) and phonon density of states (PDOS), we can effectively elucidate how the silver interface modulates phonon transport and alleviates vibrational mismatch at heterojunctions; by evaluating mean square displacement (MSD), we can reveal how the extreme spatial confinement of nanopores limits the dynamic mobility of polymer chains, thus explaining the fundamental reasons for macroscopic phase transition behavior. This is crucial for exploring the comprehensive structure-property relationships of advanced composite phase change materials.

### 3.1. Model Construction and Calculation Methods

All molecular dynamics (MD) simulations in this study were conducted using the Materials Studio (MS, 2019 version) software platform, aiming to reveal the enhancement mechanism of silver nanoparticle interface modification on the thermophysical properties of composite phase change materials at the atomic scale. Considering that the wood in the experiment had transformed into a stable carbon skeleton after high-temperature carbonization, this simulation discarded the comparison of carbonization temperature differences and instead constructed three highly representative computational models for horizontal comparison: a pure PEG model, an unsilvered carbon/PEG composite model (Unsilvered DAP), and a silvered carbon/PEG composite system (Silvered DAP).

Initially, the carbon chain skeleton and a simplified small-molecule PEG model were generated using the Amorphous Cell Calculation module. Subsequently, the composite phase change material models were constructed manually under periodic boundary conditions, introducing a metallic silver atomic layer at the contact interface between the carbon substrate and the PEG molecular chains to authentically recreate the experimentally prepared three-dimensional composite structure.

To ensure the rigorousness and reproducibility of the MD simulations, the precise physical parameters of the simulated systems were strictly defined. The PEG molecular chains were simplified to oligomers with a degree of polymerization of 5 to maintain computational feasibility while capturing the intrinsic kinetic behaviors. Specifically, the silver-containing composite system (Silvered DAP) was constructed with a physical supercell size of 32.7 Å × 32.7 Å × 129 Å. It comprises a total of 16,082 atoms, including 8113 carbon atoms, 4891 atoms constituting 130 PEG chains, and 3078 silver atoms. For comparison, the silver-free composite system (Unsilvered DAP) has a supercell size of 32.7 Å × 32.7 Å × 120 Å, comprising 18,572 atoms (12,885 carbon atoms and 5687 atoms constituting 110 PEG chains). Standard three-dimensional periodic boundary conditions (PBCs) were applied in the x, y, and z directions to eliminate vacuum edge effects and simulate a bulk-like continuous phase. Furthermore, preliminary tests with varying box dimensions confirmed that the fluctuations in the calculated interfacial thermal resistance (ITR) and thermal conductivity were well within 5%, thereby ensuring strict size convergence.

Crucially, the interfacial silver layer in the Silvered DAP model is not a simple ultra-thin layer; it possesses a robust physical thickness of 23 Å. Throughout the high-temperature simulated annealing cycles (up to 473 K) and the prolonged NPT/NVT equilibration processes, this metallic Ag layer demonstrated exceptional structural stability. The silver atoms remained robustly localized within their lattice arrangement, exhibiting no unphysical melting, mixing, or structural collapse at the heterogeneous interface.

All subsequent calculations and analyses were performed using the Forcite module. The simulations uniformly utilized the rigorously parameterized COMPASS III full-atom force field. Both electrostatic and van der Waals interactions were calculated using the atom-based summation method.

During the model optimization process, the systems first underwent geometry optimization with a maximum of 20,000 iterations to eliminate unfavorable local contacts. To achieve thorough structural relaxation, simulated annealing was performed in the NVT ensemble with a time step of 1.0 fs. This process consisted of 5 annealing cycles between 273 K and 473 K, with each cycle containing 6 heating stages, and each stage comprising 1000 dynamic steps, controlled by the Andersen thermostat.

Following annealing, a two-step dynamic equilibration was conducted. First, a 100 ps simulation was run in the NPT ensemble at 300 K and 1 atm (time step: 0.5 fs), employing random initial velocities, an Andersen thermostat, and a Berendsen barostat. Subsequently, a 100 ps simulation was performed in the NVT ensemble at 300 K, utilizing the current velocities and the Nose thermostat. Furthermore, to systematically evaluate the phase transition behavior and calculate the temperature-dependent mean square displacement (MSD), independent MD simulations were conducted across a temperature range of 278 K to 368 K at 10 K intervals, strictly adhering to the same NPT and NVT ensemble protocols. Finally, the trajectory files generated from these steps were utilized to analyze the MSD, self-diffusion coefficients, and phonon density of states (PDOS). The structural model from the final frame was extracted for thermal conductivity calculations, thereby exploring the microscopic regulatory effects of the unsilvered and silver-doped carbon skeletons on the PEG phase change materials.

### 3.2. Thermophysical and Kinetic Parameter Calculation Methods

#### 3.2.1. Mean Square Displacement (MSD) and Self-Diffusion Coefficient (D)

To characterize the migration behavior and dynamic features of PEG molecular chains within porous confined spaces, this study calculated the mean square displacement (MSD) and self-diffusion coefficient (D) based on molecular dynamics trajectories. The MSD reflects the spatial movement range of molecules within a specific time period, defined by the formula:

(1) $$\mathrm{MSD}=\frac{1}{N}\sum_{i=1}^{N}{\left|\mathrm{ri}\left(t\right)-\mathrm{ri}\left(0\right)\right|}^{2}$$

where N is the total number of atoms in the system, ri(t) is the spatial position vector of the i-th atom at time t, and ri(0) is the position vector of the atom at the initial time.

According to the Einstein relation, the self-diffusion coefficient $D$ of the system can be obtained from the slope of the MSD curve in the long-time limit:

(2) $$D=\frac{1}{2d}\underset{t\to \infty }{\lim}\frac{1}{t}\mathrm{MSD}$$

where d represents the spatial dimension of the computational system (in this study, for a three-dimensional system, d = 3).

The self-diffusion coefficient quantitatively reflects the transport capacity of particles under a unit driving force and is significantly influenced by microscopic structure and temperature. By linearly fitting the MSD curves of the system at different temperatures, the corresponding D values are calculated, and the temperature-dependent D-T curve is plotted. When the D value undergoes a sudden change as the temperature rises, this transition point marks the occurrence of a solid–liquid phase transition within the system, allowing for the precise determination of the phase change material’s melting point.

#### 3.2.2. Thermal Conductivity (TC) and Interfacial Thermal Resistance (ITR)

This study employs the non-equilibrium molecular dynamics (NEMD) method to calculate the thermal conductivity (TC) and interfacial thermal resistance (ITR) of the composite system. After energy minimization and adequate relaxation through NPT and NVT ensembles to ensure stable system density and conformation, heat source and heat sink regions are set along the axial direction (z-axis) of the model.

Specifically, the heat sources are positioned at the two periodic boundaries (ends) of the simulation box, while the heat sink is located in the central region. Driven by the Langevin thermostat, heat flows symmetrically from the ends towards the center across the periodic boundaries. Once the system reaches a steady state, this continuous bi-directional heat flux naturally generates a symmetric, V-shaped (sawtooth-like) temperature profile.

To obtain precise temperature gradients, the computational model is divided along the heat flow direction into several equally thick spatial layers (e.g., 40 layers) to compile and plot the spatial temperature distribution curve under steady state. Based on Fourier’s law of thermal conduction, linear regression analysis of the temperature gradient and heat flux density in the central heat transfer region is used to calculate the thermal conductivity Tc and interfacial thermal resistance ITR:

(3) $$\mathrm{Tc}=\frac{J}{A\nabla T}$$

(4) $$\mathrm{ITR}=\frac{∆T}{J}$$

where J represents the heat flux density during the steady-state heat transfer process, A is the cross-sectional area of the thermal conduction model, ∇T is the temperature gradient, and ΔT represents the temperature jump occurring across the multi-component interface.

#### 3.2.3. Phonon Density of States (PDOS)

To elucidate the phonon interaction pathways of heat conduction and the intrinsic origins of interfacial thermal resistance at the composite material interface from a microscopic perspective, this study further calculates the phonon density of states (PDOS). PDOS quantitatively characterizes the distribution features of phonons at different vibration frequencies. It is obtained by extracting the time-domain evolution data of the atomic velocity autocorrelation function (VACF) and performing a Fourier transform on it, with the relevant calculation formulas as follows:

(5) $$\mathrm{VACF}\left(t\right)=\frac{1}{N}\sum_{i=1}^{N}\langle \mathrm{vi}(0)\mathrm{vi}(t)\rangle$$

(6) $$\mathrm{VDOS}=\int_{-\infty }^{+\infty }e^{\mathrm{iwt}}\mathrm{VACF}(t)\mathrm{dt}$$

where vi(t) represents the instantaneous velocity vector of the i-th atom at time t.

By comparatively analyzing the overlap degree of the PDOS spectra for the carbon skeleton, silver atomic layer, and PEG molecules in the low-frequency (acoustic branch) and mid-to-high-frequency (optical branch) intervals, the molecular vibration mode matching conditions and dominant phonon scattering mechanisms at different material interfaces can be deeply explored. This provides a solid physical-level explanation for the enhancement of thermal conductivity and the evolution of interfacial thermal resistance.

## 4. Results and Discussion

### 4.1. Microscopic Morphological Features of Porous Composite Carriers

**Figure 2:**
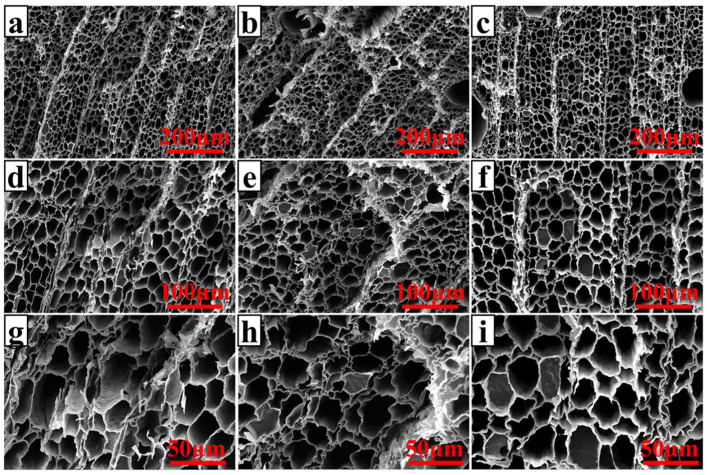
Microstructure of porous composite supports (DA) at different carbonization temperatures: (**a**,**d**,**g**) DA600; (**b**,**e**,**h**) DA800; (**c**,**f**,**i**) DA1000. From top to bottom, these represent local morphologies at low, medium, and high magnifications.

The microstructural integrity and surface modification state of the three-dimensional framework directly affect the loading and macroscopic properties of phase change materials. To investigate the influence of the carbonization final temperature on the evolution of the framework structure and the loading of nanosilver, the porous composite support after chemical silver plating was characterized at multiple scales using scanning electron microscopy, and the results are shown in Figure 2.

From the low-magnification observations (Figure 2a–c), it is visually apparent that after enduring rigorous physicochemical treatments including deep delignification, intense pyrolysis carbonization, and ultrasound-assisted wet chemical silver plating, all DA carriers perfectly retain the anisotropic honeycomb-like pores and tracheid structures of natural poplar wood. These highly interconnected and uniformly oriented micrometer-scale natural large channels did not suffer large-scale structural collapse, providing ample physical space for the subsequent vacuum impregnation of highly viscous molten PEG [19].

A lateral comparison of the morphologies at medium magnification (Figure 2d–f) reveals that different carbonization depths exert a significant regulatory effect on the shrinkage and pore size distribution of the carbon substrate. For DA600 (Figure 2d), due to the lower carbonization temperature, the skeleton shrinkage is limited. Although the pore size is larger, the graphitization degree and rigidity of the carbon cell walls are relatively insufficient. When the temperature rises to 1000 °C (Figure 2f), intense pyrolysis causes excessive removal of non-carbon elements, resulting in drastic volume shrinkage of the skeleton. The cell walls become extremely thin, and some micro-channels even exhibit a tendency to narrow under compression, which could restrict the maximum encapsulation volume of the phase change working fluid to some extent.

In contrast, DA800 exhibits the most ideal comprehensive morphology (Figure 2b,e). Under the 800 °C treatment, the carbon skeleton achieves a perfect structural balance, obtaining a robust and highly thermally conductive substrate through sufficient carbonization, while moderate volume shrinkage maintains extremely spacious and regular capillary channels. This structure not only provides excellent mechanical support but also maximizes the accommodation of PEG molecules.

Further observation of the high-magnification local morphologies (Figure 2g–i) clearly verifies the conformal growth of silver nanoparticles on the pore wall surfaces. Under the mild reduction in the silver-ammonia and glucose system, a large number of silver nanoparticles attach to the carbon substrate surface. On the inner walls of DA800 (Figure 2h), the distribution of silver nanoparticles presents excellent uniformity and density. There is neither the particle agglomeration phenomenon that might exist in some low/high-temperature samples, nor does it cause physical blockage of the micropores. Such a continuous, uniform, and highly thermally conductive metal modification layer constructed on ideal pore walls can highly efficiently bridge the thermal resistance defects inside the carbon skeleton. Based on the comprehensive advantages demonstrated by DA800 in microscopic structural integrity, pore openness, and metal network uniformity, it is foreseeable that after subsequent encapsulation of PEG, it will possess the most outstanding heat conduction and thermal energy storage potential.

### 4.2. Chemical Structure, Crystallization Characteristics, and Heat Transfer Mechanisms

**Figure 3:**
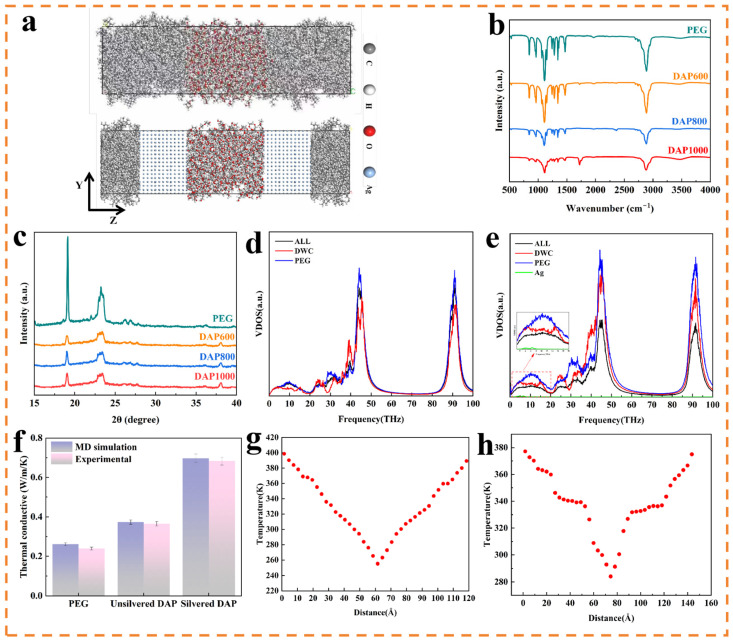
Structural characteristics and microscopic heat transfer mechanism of composite phase change materials: (**a**) The actual fully atomistic molecular dynamics models of the composite systems (Top: Silver-free composite system; Bottom: Silver-containing system clearly showing the highly dense 23 Å Ag modification layer); (**b**) Fourier transform infrared spectrum (FTIR); (**c**) X-ray diffraction pattern (XRD); (**d**,**e**) Phonon density of states spectrum (PDOS); (**f**) Comparison of experimental and simulated thermal conductivity (error bars represent the standard deviation of three independent measurements); (**g**) Interfacial temperature gradient distribution of silver-free system [18] and (**h**) Silver-containing system.

Figure 3 is systematically designed to justify the structure-property relationship of the composite materials, bridging their chemical/crystalline structural integrity with their microscopic heat transfer mechanisms. The successful encapsulation of PCMs within porous carriers and their chemical stability are foundational for ensuring the long-term application of composite materials [20,21]. The infrared spectra (Figure 3b) confirm the effective loading of PEG molecules in the porous carriers. The characteristic absorption peaks of pure PEG are completely retained in all DAP composite materials, and no characteristic peaks of new chemical bonds appear in the entire system. This indicates that the binding between the PEG molecules and the carbon skeleton modified with silver nanoparticles relies primarily on physical interactions such as capillary forces, without any chemical reactions that disrupt the original structures. The X-ray diffraction patterns (Figure 3c) further verify the crystallization state of the materials. Pure PEG exhibits sharp and strong diffraction peaks. After encapsulation into the porous carriers, the DAP samples still retain the basic diffraction characteristics of PEG, while characteristic peaks of the face-centered cubic silver crystal plane emerge in the patterns. This not only confirms the successful deposition of elemental silver but also indicates that the microscopic confined space of the carbon skeleton does not alter the basic crystalline form of PEG, thereby safeguarding the latent heat storage potential of the material.

While the aforementioned macroscopic tests confirm the structural stability, they cannot explain the fundamental physical origins of the enhanced heat conduction. To reveal the enhancement mechanism of silver nanoparticle interface modification on heat conduction performance and to properly justify the experimental results, this study conducted a quantitative analysis combining experimental testing and MD simulations, using the comprehensively optimal 800 °C carbonized sample as a representative [22]. The fully atomistic physical models (Figure 3a) visually demonstrate the realistic structural differences at the interface between the unsilvered and silvered composite systems (with the Silvered DAP model distinctly displaying the highly dense 23 Å Ag nanoparticle layer), and the thermal conductivity comparison (Figure 3f) shows that the simulated values are highly consistent with the experimental results. Test results indicate that the thermal conductivity of pure PEG is only 0.239 ± 0.05 W/(m·K). After loading it onto the unsilvered carbon skeleton (DWC800), the thermal conductivity of the composite material increases to 0.365 ± 0.05 W/(m·K). Following the in situ modification of the silver nanoparticle layer on the carbon pore walls, the experimental thermal conductivity of the optimal composite phase change material DAP800 jumps to 0.683 ± 0.05 W/(m·K). Quantitatively, this represents a remarkable 185.8% enhancement compared to pure PEG, and an 87.1% improvement over the unsilvered composite.

The significant increase in thermal conductivity stems directly from the effective reduction in ITR by the metal modification layer. The temperature gradient distributions extracted from NEMD simulations (Figure 3g [18] and Figure 3h) reveal the microscopic hindrance conditions during the heat transfer process [23]. In the unsilvered composite system, the difference in physical properties between the inorganic carbon skeleton and the organic PEG chains results in a high ITR of 4.417 × 10^−9^ m^2^·K/W, causing intense phonon scattering when heat flow crosses the interface. After introducing the silver nanoparticle layer, the ITR of the silvered system drops substantially to 2.968 × 10^−9^ m^2^·K/W [24]. This equates to a substantial quantitative reduction of 32.8% in interfacial thermal barriers. Analyzing this robust numerical correlation in conjunction with the PDOS spectra (Figure 3d,e) confirms that eliminating nearly one-third of the ITR is the fundamental atomic-level driver for the 185.8% macroscopic surge in thermal conductivity. This metal structure acts as a buffering bridge for thermal energy transmission, smoothing the phonon vibration frequency mismatch between the carbon substrate and the PEG molecules, thereby effectively suppressing interfacial phonon scattering. This microscopic dynamic mechanism profoundly explains the physical origin of the DA800 porous carrier’s ability to achieve efficient thermal energy transfer [25]. Furthermore, this multi-scale cross-validation provides a robust justification for our experimental results. The macroscopic doubling of the thermal conductivity is not merely a geometric effect of adding a highly conductive filler, but is fundamentally rooted in the atomic-level structural regulation. The uniform silver nanoparticle coating seamlessly bridges the acoustic impedance gap between the rigid carbon matrix and the flexible PEG chains, dynamically coupling the low-frequency acoustic phonons and thereby neutralizing the traditionally severe interfacial thermal resistance. This deepens the fundamental understanding of thermal transport in organic-inorganic heterogeneous confined systems.

### 4.3. Thermal Stability, Phase Change Heat Storage Performance, and Microscopic Kinetic Behavior

**Figure 4:**
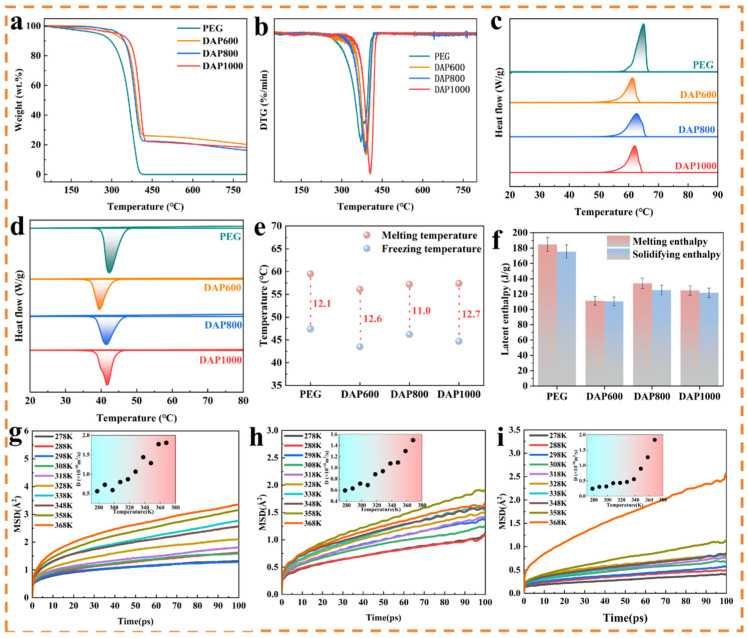
(**a**) Thermogravimetric curves and (**b**) thermal decomposition rate curves; (**c**) DSC heat flow curves for melting and (**d**) solidification processes; (**e**) phase transition temperature and (**f**) latent heat comparison; (**g**) mean square displacement (MSD) and self-diffusion coefficient (D) of pure PEG [18], (**h**) silver-free composite system and (**i**) silver-containing composite system.

Figure 4 aims to provide a comprehensive evaluation of the thermal energy storage performance by correlating macroscopic thermodynamic behaviors with microscopic molecular kinetics. Thermal stability is a core metric for evaluating the reliability of phase change materials in long-term thermal cycling applications [26]. From the TGA curve (Figure 4a), pure PEG exhibits a drastic one-step degradation process between 300 and 420 °C, which is caused by the thermal oxidative cracking of polymer segments at high temperatures. Once encapsulated in the porous carriers, the degradation behavior of the DAP composite systems shifts significantly. The thermal decomposition rate curves (Figure 4b) further reveal that the temperature corresponding to the maximum weight loss rate of the composites is markedly elevated compared to pure PEG. This enhancement in thermal stability originates from the confined space effect constructed by the three-dimensional carbon skeleton and the silver nanoparticle layer. Acting as a physical barrier, this structure effectively suppresses the thermal motion of the PEG molecular chains and delays the escape of volatile degradation products. Additionally, the residual mass percentage in the TGA curves at 800 °C accurately reflects the proportion of the carrier in the composite materials. Experimental results indicate that the loading capacities of all components are maintained at a high level, demonstrating the excellent encapsulation capability of the porous skeleton [27].

The heat storage potential of the CPCMs was quantitatively evaluated through the DSC heat flow curves (Figure 4c,d) [28]. The phase transition process of pure PEG is characterized by sharp and symmetrical endothermic and exothermic peaks, with corresponding melting and solidification latent heats of 184.8 ± 2.0 J/g and 175.3 ± 2.0 J/g, respectively. Comparing the temperature evolution trends (Figure 4e) reveals that the phase transition temperatures of the composite systems exhibit a slight decrease of 1–2 °C relative to pure PEG. This shift is generally associated with the surface tension and interfacial binding forces exerted by the porous carrier walls on the PEG molecular chains, where the spatial confinement effect of the micro/nano-channels interferes with the orderly arrangement of molecules. In the latent heat comparison (Figure 4f), although adding an inorganic carrier without heat storage activity inevitably leads to a decrease in overall energy density, DAP800 demonstrates the most outstanding heat storage performance among the composite components. Its melting and solidification latent heats are maintained at 133.9 ± 2.0 J/g and 125.2 ± 2.0 J/g, respectively. To quantitatively evaluate the energy storage effectiveness and the micro-interfacial interactions of the CPCMs, the actual PEG mass loading fraction (R) and the enthalpy efficiency (η) were systematically calculated. Based on the major weight loss platform in the TGA curve (Figure 4a), the actual loading fraction of PEG in the DAP800 composite is precisely determined to be 80.7%. Concurrently, the enthalpy efficiency, defined as the ratio of the melting latent heat of the composite to that of pure PEG ($\eta \;=\;({△H}_{m,\mathrm{CPCM}}/{△H}_{m,\mathrm{PEG}})\times 100\%$), is calculated to be 72.4%. It is scientifically noteworthy that the enthalpy efficiency (72.4%) is slightly lower than the actual mass loading fraction 80.7%. This discrepancy fundamentally originates from the micro/nano-confinement effect of the three-dimensional porous skeleton. The strong interfacial adhesion forces and the severe spatial restrictions exerted by the carbon pore walls and the dense silver nanoparticles severely hinder the free thermal mobility of the adjacent PEG molecular chains. Consequently, a fraction of the PEG segments at the heterogeneous interface is restricted from packing orderly into the crystal lattice, forming a “non-crystallizable layer” that does not contribute to the phase change latent heat. Nevertheless, successfully retaining a highly effective enthalpy efficiency of 72.4% while completely preventing liquid leakage rigorously proves the exceptional microstructural balance and encapsulation architecture of the DA800 carrier [29].

The macroscopic alterations in phase transition temperatures and latent heat observed in the DSC curves are fundamentally governed by the mobility of the polymer chains within the nanopores. To analyze the underlying causes of macroscopic phase transition behavioral changes at the molecular scale and to theoretically justify the experimental thermal shifts, this study examined the MSD and self-diffusion coefficients of different systems [30]. Observing Figure 4g [18] through Figure 4i, as temperature increases, the movement of molecular segments becomes increasingly vigorous, manifesting as a steady increase in the slope of the MSD curves. The insets showing the relationship curves between the self-diffusion coefficient D and temperature T present clear stepwise abrupt changes. These transition points accurately capture the process wherein PEG molecules break free from the constraints of the ordered crystal lattice and transform into a disordered liquid state, representing microscopic melting. Comparing Figure 4g (pure PEG), Figure 4h (unsilvered system), and Figure 4i (silvered system) reveals that the self-diffusion coefficients of PEG molecules in the composite systems are significantly lower than those in the pure phase. Moreover, the temperature offset trend of the transition points highly aligns with the phase transition temperatures measured experimentally. Quantitatively, the MSD sudden-change points in the silvered system are delayed by approximately 1.2 °C to 2.0 °C, which perfectly mirrors the macroscopic phase transition temperature shifts recorded in the DSC endothermic/exothermic peaks. This indicates that the heterogeneous interface jointly formed by the carbon skeleton and the silver nanoparticle modification layer significantly inhibits the diffusion of PEG molecular chains [31]. It not only restricts the long-range growth of crystal nuclei but also reduces the activation energy required for the cooperative motion of molecular chains through microscopic confinement effects. This dynamic analysis at the microscopic level provides a solid physical basis for the macroscopic reduction in phase transition temperature and energy storage characteristics exhibited by the DAP composites.

To further highlight the competitive advantages and practical value of the prepared CPCMs, a comprehensive comparative analysis was conducted between the optimal DAP800 sample and several recently reported state-of-the-art PEG-based composite phase change materials. The comparison systematically evaluates core thermophysical parameters, including latent heat, thermal conductivity, and leakage resistance, as summarized in Table 1.

As explicitly demonstrated in Table 1, achieving a synergistic enhancement of energy storage capacity, thermal transfer efficiency, and shape stability remains a persistent challenge. Systems with purely carbon-based porous matrices often secure high latent heat but struggle to break through the thermal conductivity bottleneck. Conversely, systems with dense, highly conductive networks often sacrifice substantial effective pore volume, leading to a precipitous drop in latent heat. In stark contrast, the DAP800 composite developed in this study exhibits distinctly superior comprehensive performance. The meticulously engineered three-dimensional carbon/silver heterogeneous interface elevates the thermal conductivity to an impressive 0.683 ± 0.05 W/(m·K), while the natural wood microchannels preserve an outstanding melting latent heat of 133.9 ± 2.0 J/g and robustly prevent liquid leakage even under prolonged thermal cycling. This comparison firmly establishes the exceptional potential of the proposed interfacial modification strategy.

### 4.4. Macroscopic Shape Stability and Thermal Response Behavior

The liquid leakage issue of organic PCMs during the solid–liquid transition is a critical bottleneck restricting their application in practical thermal management engineering [35]. To visually assess the form-stable encapsulation ability of the porous composite carriers for PCMs, all samples were uniformly placed on a 90 °C constant temperature heating stage for continuous heating over 30 min. This set temperature is far above the inherent melting point of PEG. Under this extreme condition, an infrared thermal imager was utilized to track and record the temperature distribution characteristics and macroscopic morphological evolution of the samples throughout the process.

**Figure 5:**
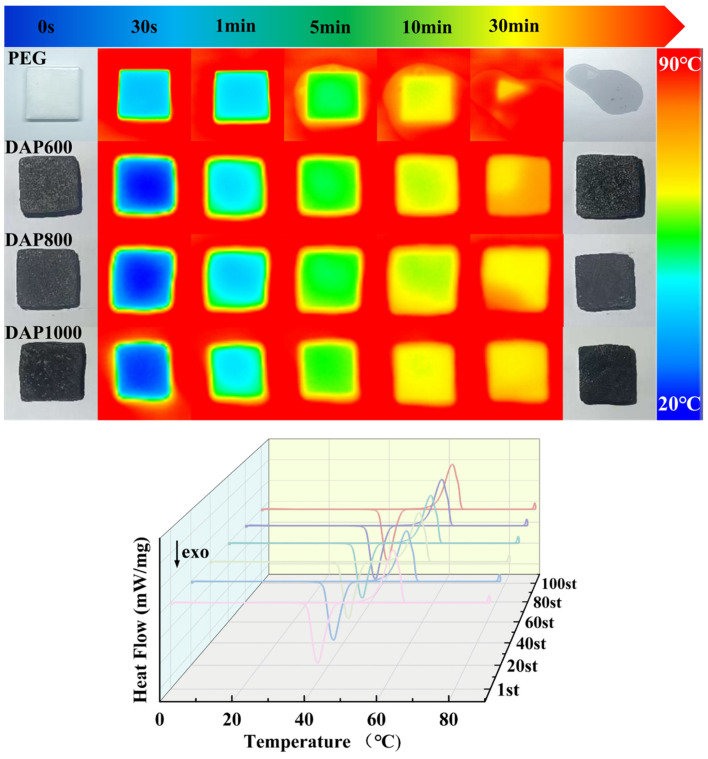
(**a**) Infrared thermal imaging and leak prevention performance testing process of pure PEG and various composite phase change materials on a 90 °C constant temperature heating stage; (**b**) DSC curves of the optimal DAP800 composite phase change material over 100 repeated melting-cooling cycles.

Observing the morphological and thermodynamic evolution trajectories in the first row of Figure 5, heat transfer within the pure PEG is slow during the initial heating stage. As heating time extends, internal phase transition occurs, and the disentanglement of molecular segments leads to a complete loss of the physical rigidity required to maintain its own shape. After heating for 30 min, the pure PEG sample has completely melted and collapsed, flowing outward in a disordered liquid state, exposing extremely poor shape stability.

In stark contrast to the complete failure of pure PEG, all DAP composite systems exhibit extremely superior leakage prevention performance. During up to 30 min of continuous high-temperature baking, the macroscopic appearances of DAP600, DAP800, and DAP1000 consistently maintain their initial regular block shapes. No signs of liquid PEG seepage are observed at the bottoms and perimeters of the samples. This excellent macroscopic shape stability directly benefits from the intricate microscopic structures inside the porous carriers. The strong capillary forces inherent to the natural directional channels of the wood, operating synergistically with the surface tension provided by the carbon skeleton and the dense silver nanoparticle modification layer, construct a formidable physical binding network at the micro and nanoscale. This network effectively overcomes the gravity and fluidity of liquid PEG, firmly locking the molten working fluid within the three-dimensional confined space, thereby thoroughly compensating for the fatal leakage defect of single PCMs [36].

Beyond verifying the anti-leakage properties, the infrared thermal image series also visually reflects the macroscopic effectiveness of the internal heat transfer networks within the materials. At identical heating time points, the surface temperatures of all DAP composite systems are notably higher than that of pure PEG, and the thermal field distribution is more uniform. Particularly within the 1 to 5 min heating interval, the surfaces of the DAP systems rapidly display a yellow-green thermal zone representing high temperatures, whereas the main body of pure PEG remains in a cool color range representing low temperatures. This agile and uniform thermal response behavior further corroborates the earlier conclusion regarding a substantial increase in thermal conductivity. The highly thermally conductive silver nanoparticles firmly attached to the pore walls couple with the continuous carbon skeleton to successfully construct high-speed, low-resistance phonon transmission channels within the composite system. This endows the materials with exceptional temperature perception and thermal scheduling capabilities.

Furthermore, to validate the long-term cyclic durability of the CPCMs in practical engineering applications, the optimal DAP800 composite was subjected to an accelerated thermal reliability test comprising 100 repeated melting-cooling cycles. As illustrated in the 3D waterfall plot in Figure 5b, the DSC curves for the 1st, 20th, 40th, 60th, 80th, and 100th cycles were systematically recorded. Remarkably, the endothermic and exothermic peaks across all tested cycles highly overlap without observable baseline drift or peak broadening. There is no significant degradation in either the phase transition temperatures or the specific latent heat enthalpy after 100 cycles. This exceptional thermal cycling reliability demonstrates that the three-dimensional carbon/silver heterogeneous skeleton not only provides a formidable physical barrier against macroscopic liquid leakage but also effectively suppresses the thermal degradation of PEG molecular chains during prolonged phase transition cycles.

### 4.5. Photothermal Conversion and Energy Storage Applications

**Figure 6:**
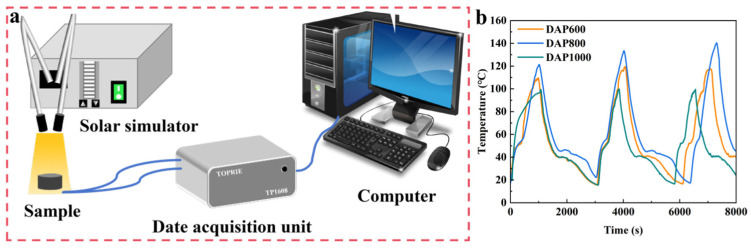
(**a**) Schematic diagram of photothermal conversion test device. (**b**) Temperature-time evolution curve under continuous illumination-cooling cycle.

Solar-driven photothermal conversion represents a critical pathway for realizing high-value applications of phase change materials [37,38]. To evaluate the solar capture and conversion efficiency of the porous composite carriers, this study built an in situ testing system comprising a solar simulator, a data acquisition unit, and a temperature monitoring terminal (Figure 6a). During testing, the simulated light source directly illuminates the surface of the composite phase change material. The generated photothermal energy rapidly transfers inward through the three-dimensional thermally conductive skeleton, driving the phase change working fluid to undergo a solid–liquid transition, thereby achieving effective thermal energy storage [39].

The temperature-time evolution curves (Figure 6b) visually document the photothermal response behavior of the materials across three continuous cycles of illumination and natural cooling. During the heating phase when the light source is activated, the surface temperatures of all DAP composite groups exhibit the typical characteristic of rapidly climbing first, followed by a fluctuating heating rate within the phase transition interval [40]. This non-linear trend occurs because the phase change material absorbs massive amounts of heat as it approaches the melting point, converting sensible heat rise into latent heat storage. After the light source is turned off, the system enters the natural cooling phase, and temperatures fall smoothly. Across three consecutive cycles, all samples maintain highly consistent heating and cooling trajectories, fully proving that this series of composite materials possesses outstanding photothermal cycling stability and energy storage reversibility [21].

By comparing the temperature response curves of different samples, it becomes evident that DAP800 displays optimal comprehensive photothermal conversion performance during cycle testing. Under identical light input conditions, DAP800 not only responds the fastest in terms of temperature rise but also achieves the highest peak photothermal temperature. This superior macroscopic performance perfectly aligns with its microscopic morphology and heat transfer mechanisms. The 800 °C carbonization treatment endows the skeleton with an appropriate porosity to ensure high-density working fluid loading, while the uniform and dense silver nanoparticle modification layer on the pore walls operates in synergy with the carbon substrate to construct an extremely low-resistance phonon transmission channel. This carbon–silver heterogeneous network efficiently absorbs photons and converts them into thermal energy, which is subsequently pumped rapidly and deeply into the internal PEG through the continuous three-dimensional thermally conductive skeleton. This effectively overcomes the sluggish thermal response defects of traditional pure polymer PCMs, maximizing the efficiency of photothermal energy capture, conduction, and storage.

## 5. Conclusions

Utilizing natural poplar wood as a precursor, this study successfully constructed a three-dimensional carbon/silver heterogeneous skeleton with directionally interconnected microchannels through delignification, gradient carbonization, and wet chemical in situ silver plating processes. Combined with the vacuum impregnation method, highly efficient form-stable encapsulation of polyethylene glycol was achieved. The study systematically explored the regulatory laws regarding the impact of carbonization temperature on the microscopic morphology and macroscopic thermophysical properties of the CPCMs. Furthermore, MD simulations were employed to deeply analyze the interfacial heat transfer and phase transition kinetic mechanisms at the atomic scale. The primary conclusions are as follows:

(1) An intermediate carbonization temperature is crucial for balancing the skeleton support strength and the working fluid loading capacity. The 800 °C carbonization treatment grants the porous carrier the most ideal structural state. While keeping the natural directional capillary channels unobstructed, it facilitates the uniform conformal growth of nano-elemental silver on the carbon pore wall surfaces. This structure provides powerful surface tension and physical binding networks at the microscopic scale, allowing the composite materials to maintain excellent shape stability even in extreme environments far exceeding the melting point, thereby completely eliminating the liquid leakage of the phase change working fluid.
(2) Silver nanoparticle interface modification significantly enhances the thermal energy transfer efficiency of the composite system. Experiments measured the thermal conductivity of the optimal sample DAP800 to be 0.683 ± 0.05 W/(m·K), which is nearly a two-fold increase over the pure PCM. MD simulations and PDOS analyses confirm that the high-density metallic silver modification layer serves as a buffering bridge for thermal energy transmission. It effectively smooths the phonon vibration frequency mismatch between the inorganic carbon substrate and the organic polymer, thereby substantially reducing interfacial thermal resistance and suppressing phonon scattering.
(3) The composite phase change materials demonstrate outstanding heat storage capabilities and comprehensive application potential. DAP800 maintains an excellent melting latent heat of 133.9 ± 2.0 J/g. Through MSD analysis, the simulations revealed the kinetic insight that the nano-confined space significantly restricts molecular chain mobility, which effectively regulates the phase transition activation energy of the molecules and perfectly explains the experimentally observed thermal shifts. In continuous illumination and natural cooling cycle tests, the material exhibited agile thermal response behavior and exceptional photothermal conversion reversibility. This research not only provides detailed experimental data for the application of wood-derived porous materials in advanced thermal energy storage fields but also offers vital theoretical guidance for designing high-performance photothermal conversion and thermal management devices based on multi-scale interface engineering.

## Figures and Tables

**Table 1:** Comparison of thermophysical properties between the prepared DAP800 and reported PEG-based and other typical composite phase change materials.

Material	PCM	Latent Heat (J/g)	Thermal Conductivity (W/(m·K))	Leakage Resistance	Reference
GF@PPy	PEG	158.5	0.415	Excellent	[32]
BPC@Ag	PEG	134.1	0.68	Excellent	[9]
Cu@rGO-CMF	PEG	149.8	0.45	Excellent	[33]
Ag@rGO-CMF	PEG	154~157.7	0.49	Excellent	[15]
PVA/graphene	PW	165.3	0.486	Excellent	[34]
Wood-derived porous carbon	PEG	133.9 ± 2.0	0.683 ± 0.05	Excellent (100 cycles)	This work

## Data Availability

The original contributions presented in this study are included in the article. Further inquiries can be directed to the corresponding author.
